# Myokines: Novel therapeutic targets for diabetic nephropathy

**DOI:** 10.3389/fendo.2022.1014581

**Published:** 2022-10-25

**Authors:** Ming Yang, Shilu Luo, Jinfei Yang, Wei Chen, Liyu He, Di Liu, Li Zhao, Xi Wang

**Affiliations:** ^1^ Department of Nutrition, Xiangya Hospital, Central South University, Changsha, Hunan, China; ^2^ Department of Nephrology, The Second Xiangya Hospital of Central South University, Changsha, China; ^3^ Department of Reproduction and Genetics, The First Affiliated Hospital of Kunming Medical University, Kunming, China; ^4^ National Clinical Research Center for Geriatric Disorders, Xiangya Hospital, Central South University, Changsha, China

**Keywords:** myokines, diabetic nephropathy, muscle, irisin, FGF21

## Abstract

With the increasing incidence of diabetic nephropathy (DN), there is an urgent need to find effective DN preventive and therapeutic modalities. It is widely believed that effective exercise is good for health. However, the beneficial role of exercise in kidney disease, especially in DN, and the underlying molecular mechanisms have rarely been reported. Muscle is not only an important motor organ but also an important endocrine organ, secreting a group of proteins called “myokines” into the blood circulation. Circulating myokines then move to various target organs to play different biological roles. In this review, we summarize the currently known myokines and the progress in research relating them to DN and discuss its potential as a therapeutic target for DN.

## Introduction

Diabetic nephropathy (DN) has been the main cause of end-stage renal disease (ESRD) in developed countries. With social-economy development of and the improvement of people’s living standards, DN incidence has increased drastically over the past two decades (1–3). DN is often accompanied by severe retinopathy (4, 5), neuropathy (6, 7), cardiomyopathy (8, 9), and other complications. Therefore, there is an urgent need to find effective preventive and therapeutic modalities for DN. Recently, research on the treatment of DN has focused on inter-organ crosstalk.

**Figure 1:**
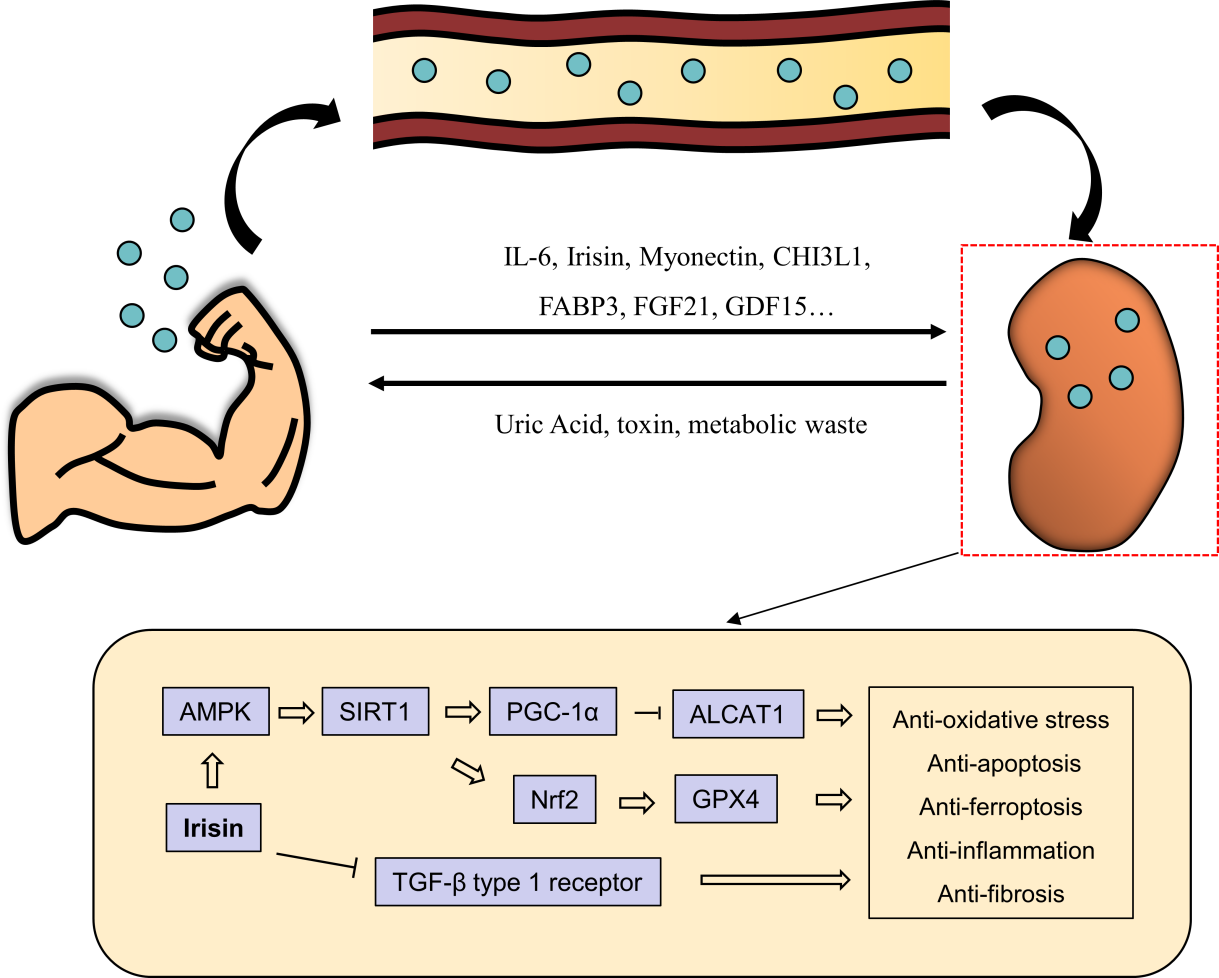
The muscle-renal axis. Effective exercise increases the production of myokines, such as IL-6, irisin, myonectin, FABP3, FGF21 and GDF15, from muscles and their secretion into the blood circulation. Myokines then travel through the bloodstream to the kidneys, among other organs, where they exert various biological effects. For example, irisin exerts renoprotective effects through different pathways. It can activate AMPK/SIRT1/PGC-1a pathway to inhibit ALCAT1 expression and thus anti-apoptosis, and activate GPX4 through AMPK/SIRT1/Nrf2 pathway to inhibit ferroptosis.

Muscle is the largest tissue in the body, accounting for approximately 30% - 40% of the total body weight (10). Muscle weight is affected by many factors, such as diet, chronic diseases, and tumors (11). In humans, muscles are of various different types, including skeletal muscle, cardiac muscle, and smooth muscle, depending on their position and function. These are responsible for maintaining the human body’s movement and balance, producing heat and the mechanics internal organs, such as the heart, digestive organs, and blood vessels (12). It is well known that exercise is good for human health. Recent research has revealed that muscles can secrete a group of proteins called “myokines” in response to external stimuli to participate in the maintenance of homeostasis. Myokines are secreted by muscles into the blood, from where they are circulated to other cells, tissues, and organs to perform various functions (13–16) (**Figure 1**). In this review, we summarize the currently discovered myokines and the progress in research relating them to DN and we also discuss its potential as a therapeutic target for DN.

## Myokines and DN

### Interleukin-6

Interleukin-6 (IL-6) is a cytokine that plays an important role in inflammation, immune response and hematopoiesis (17, 18). The relationship between IL-6 and muscle was found in 1998 when Ostrowski et al. demonstrated that blood IL-6 levels increased significantly after exercise (19) and its levels increased in preference to those of other cytokines (20). After exercise, plasma IL-6 levels can be more than 100 times higher than those at rest (21, 22); after an ultramarathon, plasma IL-6 levels can even rise to an astonishing 8,000 times those at rest (23). It was believed that such an increased in IL-6 levels after exercise is thought to be a consequence of muscle damage. Pedersen et al. showed that the increase in plasma IL-6 levels after exercise did not result from muscle injury but rather from the intensity of exercise (20). Moreover, exercise involving only a few muscles is not enough to increase plasma IL-6 levels (24–26); only exercise involving all muscles, such as running, can significantly increase plasma IL-6 levels. Additionally, the expression of IL-6 mRNA was found to be low in muscles during rest, but it increased to more than 100 times the level at rest after exercise (27, 28). These findings suggest that muscle contraction during exercise releases large amounts of IL-6, increasing its plasma levels. Interestingly, several studies have shown that more physical activity is associated with lower levels of plasma IL-6 (29–31).

Several studies have provided insight into the role of IL-6 in DN, which is a metabolic inflammatory disease. Senthilkumar et al. demonstrated that the levels of IL-6 and insulin were notably increased in the serum of type 2 diabetes mellitus (T2DM) patient patients with DN compared to those in T2DM patients without DN (32). Similarly, plasma IL-6 levels were elevated in the early stages of CKD but not further up-regulated with progression to in the more severe stages of CKD (33). The levels of IL-6 and other inflammatory factors were also increased in mice with STZ-induced DN (34). Another report revealed that miR-34b could relieve inflammation and apoptosis of HK-2 cells under high glucose intervention by inhibiting the IL-6R/JAK2/STAT3 signaling pathway (35). Systemic inflammation is aggravated in DN, and the resulting increase in IL-6 levels further aggravates DN progression. Conversely, inhibition of IL-6 can alleviate kidney damage caused by high glucose. These findings indicating that intense exercise leads to high plasma IL6 levels seem to contradict the idea that exercise is good for health. However, it may be that exercise only temporarily increases plasma IL-6 levels, while significantly decreasing baseline plasma IL-6 levels (29, 30). Prolonged low baseline plasma IL-6 levels may alleviate systemic inflammation and thus delay the progression of DN.

### Irisin

In 2012, Boström et al. showed that transgenic mice overexpressing peroxlsome proliferator-activated receptor-γ coactlvator-1α (PGC1α) had increased production of FUNDC5 protein in their muscle (36). FUNDC5 contains a signal peptide, a fibronectin III domain, a hydrophobic transmembrane domain, and a carboxy-terminal domain (37). After a series of processing and modification steps, such as proteolytic lysis and glycosylation, FUNDC5 was reconstituted as a new protein mainly containing the fibronectin III domain called irisin (38–40). Irisin is made up of 112 amino acids and its amino acid structure is identical between humans and mice (41). In humans, FUNDC5 is mainly expressed in muscle and adipose tissue and has low expression in the pancreas and liver. Muscle is the main source of circulating irisin. In mice, muscle-derived irisin accounts for about 78% of the total circulating irisin; the remaining 22% may be derived from adipose tissue (37). Exercise was shown to be closely related to irisin secretion from muscle. Boström et al. demonstrated that mice that ran regularly for three weeks had a 65% increase in irisin levels compared to control mice (36). Huh et al. observed that circulating irisin levels in untrained healthy women who underwent whole-body vibration training increased by 9.5% and 18.1% at week 0 and 6, respectively; six weeks of training did not change circulating irisin levels in the resting state, but it significantly increased the magnitude of the training-induced increase in irisin levels (42). Similarly, single high-intensity endurance training and high-intensity training led to a temporary increase in circulating irisin levels, which peaked at 1 h and then gradually decreased to the baseline level (43). These findings strongly indicate that exercise can promote irisin secretion from muscle into the blood circulation, from where irisin moves to target organs to play its physiological role in regulating body homeostasis. Research regarding receptors of irisin on the surface of cells or tissues is still controversial. Kim et al. demonstrated that αV integrin receptors may act as the receptors of irisin to mediate signal transfer into cells; treatment of cells with the integrin inhibitor RGD peptide could partially inhibit the effect of irisin (44). Peng et al. showed that irisin could also reduce renal tubular cell injury by inhibiting the TGF-β type 1 receptor (45). However, the receptor repertoire of irisin needs to be further elucidated in the future to clarify the mechanisms underlying irisin effects.

The role of irisin in DN has been partially revealed. Wang et al. showed that, compared with T2DM patients with normal albuminuria, the level of serum irisin was significantly reduced in patients with microalbuminuria and macroalbuminuria (46). In addition, with the increase in proteinuria and decrease in the of glomerular filtration rate, the serum irisin level further decreased (46). Moreover, Mageswari et al. showed that the circulating irisin level was notably increased in patients with DN compared with diabetic patients without nephropathy and was strongly associated with eGFR (47). These findings suggest that serum irisin may be an indicator of DN progression; Some studies have focused on the researching the mechanism underlying the role of irisin in DN progression. In addition, aerobic physical exercise reduced albuminuria, glomerular hypertrophy and inflammation in the kidney of rats compared to sedentary diabetic rats, while these beneficial effects of exercise were blocked by treatment of CycloRGDyK, an irisin receptor blocker (48). In mice with ischemia reperfusion (I/R)-induced acute kidney injury (AKI), irisin could protect renal mitochondrial function and reduce oxidative stress and inflammation by up-regulating the expression of GPX4 (49). What’s more, Wu et al. showed that aerobic exercise could activate the irisin-AMPK-SIRT1-PGC-1α signaling pathway, inhibiting the expression of ALCAT1 and ultimately improving the oxidative stress level and apoptosis caused by kidney injury following myocardial infarction (50). Moreover, muscle-specific overexpression of PGC-1α promoted the secretion of irisin, inhibiting the activation of the TGF-β type 1 receptor in renal tubular cells and ultimately improving renal energy metabolism and inhibiting renal fibrosis (45). In addition to renoprotective effects, irisin could alleviate vascular calcification in chronic kidney disease by maintaining mitochondrial function or inhibiting pyroptosis (51). These studies suggest that irisin could be considered to be used as a potential target for kidney therapy in the future.

### Myonectin

Myonectin is a myokine recently reported in 2012 by Seldin et al. (52) and Lim et al. (53). It’s a Member of the C1q/TNF-related protein (CTRP) family. Myonectin is mainly expressed and secreted by muscle tissue and mediates the exchange of signals between muscles and other metabolic organs, such as adipose tissue and liver, to coordinate metabolic levels throughout the body (54, 55). The expression of myonectin is regulated by exercise and nutritional status. Otaka et al. demonstrated that mice treated with treadmill exercise had a significantly up-regulated myonectin level in their serum compared to control mice. In addition, myonectin was notably increased upon treatment of myotubes cultured in serum-free medium with glucose or free fatty acid; Similarly, myonectin was significantly increased in mice fed a diet with high glucose and fat after a night of fasting (52). Myonectin could stimulate the lipid uptake capacity of adipose tissue and liver by up-regulating proteins involved in lipid uptake (CD36, FATP1, Fabp1 and Fabp4) and, thereby, reduce circulating free fatty acids levels without significantly affecting the lipolysis and glucose homeostasis of adipose cells (52, 54). Therefore, myonectin may act as a nutrition-sensing factor that can timely transmit information about the nutritional status of the body to various tissues.

There are few studies focusing on the role of myonectin in DN, a disorder of nutrient metabolism. A clinical study revealed that the serum myonectin levels in patients with DN were lower than those in control patients; moreover, the serum myonectin levels were further reduced in the macroalbuminuria group compared to those in the normoalbuminuria and microalbuminuria groups. Logistic regression analysis showed an association between myonectin level and a lower risk of T2DM and DN (56). Few studies have explored the potential mechanism through which myonectin acts in renal disease. Renal lipid metabolism disorder and renal ectopic lipid deposition are important clinical manifestations of DN as well as key factors involved in the aggravation of DN progression. Considering the central role of myonectin in regulating lipid metabolism, these processes may form the link between myonectin and DN.

### Chitinase-3-like protein 1 (CHI3L1/YKL-40)

Glycoside hydrolase family 18 consists of chitinases and non-enzymatic chitinase-like proteins (CLPs), both of which can bind chitin (57). Chitinase-3-like protein-1 (CHI3L1) is a CLP and is called YKL-40 in humans (58–60). Görgens et al. demonstrated that CHI3L1 is a myokine, the levels of which are significantly upregulated in muscles and serum by exercise [59]. Electrical stimulation of cultured myotubes can also significantly increase *CHI3L1* mRNA expression and CHI3L1 secretion (61). A similar finding was that the expression of CHI3L1 was notably increased after 1 h of exercise and increased further after 3 h of exercise (62). After secretion into the blood circulation, CHI3L1 can move to target organs to promote cell proliferation, differentiation and anti-apoptosis (63).

Røndbjerg et al. revealed the relationship between CHI3L1 and DN. They divided 105 patients with T2D into normal albuminuria, persistent microalbuminuria, and persistent macroalbuminuria groups according to their amount of urinary protein; 20 healthy people were placed in the control group. Serum YKL-40 levels were significantly increased in the group with persistent proteinuria compared with those in the other groups and correlated with the urinary albumin/creatinine ratio (64). Similarly, the level of YKL-40 was found to be increased in the early stage of DN and was correlated DN progression (65, 66). These findings suggest that YKL-40 may be used as a diagnostic indicator for early-stage of DN. However, whether the level of YKL-40 in DN is increased secondary to its decreased excretion through the kidney, and its role in renal injury in DN remain to be further studied.

### Fatty acid-binding protein 3

Fatty acid-binding proteins (FABPs) are an intracellular group of proteins, with a molecular weight of 14-15 kDa and 126-134 amino acids, that participate in the regulation of intracellular lipid metabolism (67). At present, there are nine known FABPs. The amino acid sequences of different FABPs have 20-70% homology as well as some similarities in spatial structure. Among the FABPs, FABP3 is the most widely distributed, mainly in muscle, kidney, lung, brain, and ovary tissues (68). The level of FABP3 is regulated by many factors, and exercise is one of the more critical factors affecting it. Hutchinson et al. demonstrated that non-pregnant women who performed moderate-intensity treadmill walks (40-60% of heart rate reserve) had significantly increased FABP3 levels than resting women (69). Similarly, the secretion of FABP-3 from rat gastrocnemius also increased during exercise (70). Studies have revealed that FABP3 is also involved in the progression of many diseases. The knockout of FABP3 aggravated transverse aortic constriction-induced cardiac hypertrophy and cardiac insufficiency. A multi-omics analysis revealed that FABP3 knockdown induced cardiac dysfunction with increased glycolysis, lipid accumulation, impaired fatty acid oxidation, and decreased ATP synthesis under hypertrophy (71). Mechanically, FABP3 mediates cardiometabolic reprogramming by directly interacting with PPARα (71). Moreover, the downregulation of FABP3 expression can lead to changes in mitochondrial morphology, decrease intracellular ATP synthesis, increase mitochondrial ROS production, and ultimately induce apoptosis (72).

Unfortunately, few studies have explored the involvement of FABP3 in DN. Ozawa et al. showed, through a microarray assay with isolated glomeruli, that the mRNA expression of FABP3 in glomeruli was significantly increased in the kidneys of diabetic eNOS knockout mice compared to that in the kidneys of control group mice, and that this high expression of FABP3 was associated with MCP-1 expression and renal infiltration of CD68 cells (73). Furthermore, FABP3 levels were found to be significantly increased in the urine of patients with diabetes prior to the occurrence of microalbuminuria (73, 74). Similarly, Yu et al. demonstrated that the FABP3 level was up-regulated in parallel with the eGFR level in patients with diabetes, and this increase in the FABP3 level was independently and significantly correlated with eGFR stages G2-G4 (75). In one study, urine and serum samples were collected from 120 patients with AKI to assess the need for timely initiation of renal replacement therapy (RRT). Urinary proteomics showed that increased urinary FABP3 levels can serve as a diagnostic/prognostic indicator of RRT initiation in AKI patients (76). These findings suggest that FABP3 levels in the blood and urine are significantly increased in DN or AKI and may play an adverse role in the progression of kidney disease. This suggestion again goes against our belief that exercise is good for health. However, further studies will we need to rule out whether the above mentioned are compensatory increases in FABP3 levels in response to kidney injury or whether they are of non-muscular origin. Although the specific mechanism needs to be further studied, current studies suggest that FABP3 may be an important predictor of DN.

### FGF21

The fibroblast growth factor (FGF) superfamily is composed of 23 polypeptides, which play their roles mainly in autocrine or paracrine form (77). However, FGF15/19, FGF21, and FGF23 are released into the blood circulation and act on target organs in an endocrine manner. Paracrine and endocrine FGF signaling is mediated primarily by the activation of FGF receptors (FGFRs), including FGFRs 1b, 1c, 2b, 2c, 3b, 3c and 4. When FGF binds to its receptors, it mediates intracellular signaling through four pathways: RAS/RAF/mitogen-activation protein kinase (MAPK) signaling pathway, phosphatidylinositol 3-kinase (PI3K)/serine-threonine protein kinase AKT signaling pathway, signal transducer and activator of transcription (STAT) signaling pathway, and phosphoinositide phospholipase C (PLC) γ signaling pathway (78, 79). FGF21 contains 209 and 210 amino acid residues in humans and rodents, respectively; it consists 13 N-terminal residues and 40 C-terminal residues and the gene encoding FGF21 is located on chromosome 19 (80). The activation of the FGFR1 receptor by FGF21 requires the involvement of the cofactor β-klotho (81). As a regulatory metabolic molecule, FGF21 plays an important role in maintaining metabolic homeostasis. One study showed that FGF21 inhibited glucolipid-induced islet cell apoptosis, promoted beta cell survival and function, and increased the number of insulin-positive islets in dB/dB mice, thereby contributing to the maintenance of glucose homeostasis and prevention of hyperglycemia (82). Conversely, the absence of FGF21 resulted in insulin resistance and islet dysfunction (83, 84). The expression of FGF21 is mainly regulated by metabolic factors, including fasting (85), a high carbohydrate diet (86), and low protein diet (87). FGF21 is mainly expressed in the liver and pancreas (84, 88, 89), but recent studies have confirmed that it is also a myokine and can, therefore, also be secreted into the blood by muscles. Hojman et al. demonstrated that the expression of FGF21 in the muscles of normal young men was low but significantly increased 3 or 4 h after insulin injection along with an increased in plasma FGF21 levels (90). There has also been increasing evidence indicating that muscle tissue is an important source of circulating FGF21 (91).

Several studies have confirmed that FGF21 plays an important role in the progression of DN. El-Saeed et al. observed that the serum FGF21 levels in diabetic patients with normoalbuminuria were significantly increased compared to those in the control group patients, and were positively correlated with cholesterol, triglyceride, LDL cholesterol, creatinine, HA1C, UAE, and other biochemical indexes while being negatively correlated with glomerular filtration rate (92). Similarly, with the increase in the proteinuria level, the serum FGF21 level in patients with DN also increased significantly compared to that in the control group patients (93–96). Lin et al. demonstrated that treatment with FGF21 could significantly relieve renal tubulointerstitial lesions and fibrosis by activating the AKT/MDM2/p53 signaling pathway and inhibiting TGF-β/Smad2/3-mediated epithelial-to-mesenchymal transition in the kidneys of mice with DN (97). Moreover, fenofibrate, a commonly used lipid-lowering drug, is used to treat hyperlipidemia in patients with DN. However, recent studies have shown that, in addition to its role in improving DN through lipid-lowering, fenofibrate can also play a renal protective role directly through FGF21. Cheng et al. showed that fenofibrate plays a renal protective role in DN by promoting the expression of FGF21 and thereby activating the Akt2/GSK-3β/Fyn/Nrf2 antioxidant and AMPK pathway (98). The benefits of FGF21 in combination with other drugs in the treatment of DN have also been revealed. Meng et al. showed that, compared with insulin or FGF21 alone, FGF21 combined with insulin can further improve blood glucose levels and renal pathological changes, oxidative stress, and AGEs caused by high glucose in mice with DN (99). Mechanically, these effects may occur through the promotion of AMPK phosphorylation and inhibition of mTOR phosphorylation (99). In addition, treatment with low-dose radiation (LDR) in combination with FGF21 significantly reduced diabetes-induced renal fibrosis, inflammation, and oxidative damage in mice with STZ-induced DN compared with LDR and/or FGF21 alone (100). These findings strongly suggest that FGF21 is closely related to the progression of DN and can be used as a therapeutic target of DN.

### Growth differentiation factor 15 (GDF15)

GDF15 is a member of the cell stress–responsive transforming growth factor-β (TGFβ) family, also known as macrophage inhibitory cytokine-1 (MIC-1) and NSAID activated gene 1 (NAG-1) (101, 102). GDF15 is a monomeric precursor protein with a molecular weight of about 40 kDa. After dimerization, GDF15 is cleaved to form a mature dimer with a molecular weight of 30 kDa (103). GDF15 is a stress-inducing hormone and is released by cells, such as vascular smooth muscle cells, heart and endothelial cells, macrophages, and fat cells in response to external stimuli resulting from various stress states, such as obesity, insulin resistance, heart failure, and cancer (104–108). Compared with those of other cytokines, the basal level of GDF15 in serum is higher in the resting state, 0.2-1.2 ng/mL (103, 109), and gradually increases with age. Moreover, exercise is a key factor causing increased GDF15 levels in the blood circulation. Klein et al. showed that prolonged endurance exercise induced a 4-5-fold increase in circulating GDF15 in mice and humans compared to respective controls. Interestingly, the pharmacological inhibition of GDF15 suppressed voluntary running in mice (110). Similarly, in marathon runners, GDF15 levels in the blood circulation increased significantly immediately after the race and returned to basal levels within 48 h (111). After exercise, the *GDF15* mRNA level in the soleus muscle of mice was increased (110). Moreover, electrical stimulation induced the contraction of primary human muscle cells for 3 h and also induced the release of GDF15 into the culture medium (112). These findings suggest that exercise can induce muscle to release GDF15; however, the underlying mechanism and events downstream of GDF15 secretion into blood remain to be further investigated.

As a nutrient-sensing and regulatory factor, GDF15 plays a key role in metabolic diseases, such as obesity and diabetes. However, the relationship between GDF15 and DN is rarely studied. Lajer et al. revealed that the level of GDF15 was elevated in type 1 DN and higher GDF-15 levels were associated with faster deterioration of renal function (113). Moreover, GDF-15 was found to be a predictor of all-cause and cardiovascular mortality and morbidity in patients with DN (113). Similarly, Ho et al. found that higher plasma GDF-15 levels are associated with microalbuminuria and predict the incidence of CKD (114). Moreover, the T2D patients with a lower-than-normal glomerular filtration rate had up-regulated urine GDF15 levels compared to the control patients (115). These findings indicate that GDF15 plays a detrimental role in the progression of DN. However, the increased level of GDF15 in DN may be caused by a variety of factors. One such factor is the increased level of GDF15 production in the body. In addition, due to the impaired renal function in DN, reduced excretion of GDF15 leads to its increased retention. In the future, more experiments are needed to reveal the role of GDF15 in the progression of DN and its underlying molecular mechanism.

In addition to what we have summarized above, with the development of biotechnology, more and more myokines have been discovered, such as Apelin (116), Chitinase-3-like protein (117), Follistatin-like 1 (118), Dipeptidyl Peptidase IV (119), MG53 (120) and METRNL (121), which they also play a key role in maintaining the human homeostasis of the body. Further studies are needed to determine whether they are related to DN and, if so, how **Table 1**.

**Table 1:** Some myokines as currently defined.

Myokines	Biological effects	References
Irisin	Anti-oxidative stress, anti-apoptosis and anti-fibrosis	(48, 122)
Myonectin	Regulating lipid metabolism	(123)
CHI3L1	Anti-apoptosis	(57)
FABP3	Regulating lipid metabolism	(71)
FGF21	Anti-oxidative stress, anti-apoptosis and anti-inflammation	(124, 125)
GDF15	Regulating metabolic homeostasis	(126, 127)
Apelin	Increased Insulin sensitivity	(128)
FSTL-1	Anti-oxidative stress, anti-apoptosis and anti-inflammation	(63, 129)
DPP-IV	Regulating metabolic homeostasis	(119)
MG53	Regulating insulin sensitivity	(130, 131)
METRNL	Anti-inflammation and regulating insulin resistance	(132)

## Conclusion and future prospects

The role of abnormal signaling between organs of the human body in the occurrence and development of diseases has gradually attracted the attention of researchers. It is widely believed that effective exercise is good for health; however, exact molecular mechanisms underlying the effects of exercise on human health are still unclear. Here, we have summarized reports indicating that muscle, as an endocrine organ, regulates systemic metabolism and participates in the progression of diseases by secreting different kinds of myokines. We have also discussed the roles of these myokines in DN. Some myokines are secreted by non-injured muscles after exercise, while others are leaked into the circulation after muscle injury. These myokines play different roles as therapeutic targets and predictors of DN. Unfortunately, the functional decline of kidneys, an important excretory organ of the human body, in DN leads to the failure of muscle factor discharge through urine in time. This impact the way we study the effect of myokines on kidneys in DN. Therefore, we can expect the “muscle-renal axis” may serve as a target for the prevention and treatment of DN in the near future.

## Author contributions

MY and XW wrote the manuscript, SL, JY, WC, LH, DL, and LZ edited the manuscript. All authors read and approved the final manuscript.

## Funding

This work was supported by the National Natural Science Foundation of China (81900069, 82000697).

## Acknowledgments

We thank Bullet Edits Limited for the linguistic editing and proofreading of the manuscript.

## Conflict of interest

The authors declare that the research was conducted in the absence of any commercial or financial relationships that could be construed as a potential conflict of interest.

## Publisher’s note

All claims expressed in this article are solely those of the authors and do not necessarily represent those of their affiliated organizations, or those of the publisher, the editors and the reviewers. Any product that may be evaluated in this article, or claim that may be made by its manufacturer, is not guaranteed or endorsed by the publisher.
